# Examining the Prevalence of Congenital Anomalies in Newborns: A Cross-Sectional Study at a Tertiary Care Maternity Hospital in Saudi Arabia

**DOI:** 10.3390/children11020188

**Published:** 2024-02-02

**Authors:** Bayapa Reddy Narapureddy, Yousef Zahrani, Hind Eissa Musa Alqahtani, Bharat Kumar Mamilla Mugaiahgari, Lingala Kalyan Viswanath Reddy, Shaik Mohammed Asif, Mohammad Ali Abdullah Almoyad, Ali Mohieldin, Adam Dawria

**Affiliations:** 1 Department of Public Health, College of Applied Medical Sciences, Khamis Musait, King Khalid University, Abha 61421, Saudi Arabia; y.zahrani@kku.edu.sa (Y.Z.); almoibrahim@kku.edu.sa (A.M.); aabdelqader@kku.edu.sa (A.D.); 2 Maternity and Children Hospital, Ministry of Health, Al Mahala, Abha 61421, Saudi Arabia; healgahtani@moh.gov.sa; 3 Department of Basic Medical Sciences, College of Applied Medical Sciences, Khamis Musait, King Khalid University, Abha 61421, Saudi Arabia; bmugaiahgari@kku.edu.sa (B.K.M.M.); maabdulllah@kku.edu.sa (M.A.A.A.); 4 Department of Public Health, College of Health Sciences, Saudi Electronic University, Abha 61421, Saudi Arabia; lv.reddy@seu.edu.sa; 5 Department of Diagnostic Science and Oral Biology, College of Dentistry, King Khalid University, Abha 62529, Saudi Arabia; mshaik@kku.edu.sa

**Keywords:** congenital anomalies, prevalence, neonatal outcomes, consanguinity, risk factors, genetic counseling

## Abstract

**Background:** Congenital anomalies, representing structural or functional abnormalities present at birth, pose a substantial global health challenge, affecting 8 million newborns annually. With 3.3 million succumbing before age five and 3.2 million facing physical or mental disability, their diverse causes necessitate comprehensive understanding for effective healthcare planning. This study explores the prevalence of congenital anomalies among newborns in the Abha Maternity and Children Hospital (MCH) in Abha, Kingdom of Saudi Arabia. **Methodology:** A descriptive cross-sectional record-based study was conducted on newborns born between 2018 and 2022. Data were gathered in 4 months from September to December 2023. Purposive sampling was employed to select the case records of newborns with congenital anomalies after careful screening and considering inclusion and exclusion criteria. Data was acquired through a self-designed study tool, and the data were entered into Google Forms. **Results:** Congenital anomalies’ five-year prevalence was 3.21%, and one year, in 2022, the prevalence was 4.02%. Female neonates exhibited higher anomalies (59.3%), and preterm births accounted for 39.6%, emphasizing their vulnerability. The findings indicate that consanguineous marriages are linked to 63.3% of anomalies, notably neural tube defects (25%) and congenital heart diseases (19.7%). Anomalies are not significantly associated with consanguinity or birth order, but maternal age, education, employment, and antenatal maternal medical issues are associated considerably. **Conclusions:** These study insights contribute to health planners planning targeted interventions and awareness programs that are crucial to mitigate risks associated with preterm births and consanguineous marriages. The promotion of 100% antenatal screening and prophylactic medication for high-risk women and couples is necessary to prevent inherited deformities. In future the Ministry of Health must plan large-group prospective research to better understand the associated risk factors that will help public health initiatives minimize congenital-associated neonatal mortality and improve pregnancy outcomes.

## 1. Introduction

A congenital anomaly (CA) refers to an abnormality present at birth, affecting function, structure, or metabolism, potentially leading to physical or mental disabilities or even fatalities. These anomalies can severely impact organs, limbs, and any system in the body [1]. CA affects individuals and their families with long-term physical, economic, psychological, and social consequences [2]. Globally, eight million children are born with congenital anomalies each year, resulting in 3.3 million deaths before they reach their fifth birthday and 3.2 million survivors facing potential physical or mental disabilities [1,3].

The causes of congenital anomalies include nutritional, genetic, environmental, economic, or multifactorial factors, with environmental pollutants, drugs, and infectious agents being significant concerns. Understanding the interplay of genetic, environmental, socioeconomic, and demographic factors is essential for addressing congenital anomalies. While ecological pollutants, drugs, and infectious agents pose global concerns, there is still a lack of clarity regarding the underlying causes of many congenital anomalies, emphasizing the complexity often attributed to multifactorial inheritance in typical cases. Effective healthcare planning and prevention strategies hinge on unravelling these complexities [2,4]. Understanding the occurrence and trends of congenital malformations is crucial for identifying factors that may cause or prevent them. The timely antenatal detection of significant congenital anomalies is essential for potential pregnancy termination, either fetal or neonatal. The extent of substantial congenital anomalies in Saudi Arabia has not been thoroughly explored [1].

Approximately 15% to 25% of congenital anomalies stem from recognized genetic conditions, 8% to 12% stem from environmental factors, and 20% to 25% stem from multifactorial like socio-demographic and economic factors [1,5]. However, the majority (40% to 60%) remain unexplained [4].

In Saudi Arabia, recent studies estimate that the major and minor congenital malformation prevalence was 27.1/1000 live births, with the highest rates in cardiovascular (7.1/1000) and musculoskeletal/limb malformations (4.1/1000) [1]. Other studies found at birth prevalence of CAs was 41.2/1000 live births [6] and 23/1000 live births. [7]. Gastrointestinal malformations were 1.3 per 1000 live births, and neural tube defects were 1.9 per 1000 live births [8,9].

Congenital anomalies globally emerging as a potential public health problem contribute to neonatal and infant mortality, long-term disabilities, and higher healthcare costs [10]. The registration and monitoring of congenital anomalies are vital for identifying clusters and trends. Early prenatal diagnosis is crucial for counselling, intervention, and possible fetal therapy. Assessing their prevalence in Saudi Arabia is vital for informed healthcare decisions. This study, considering its cultural and ethnic diversity, aimed to provide a nuanced understanding of the prevalence of congenital anomalies and the complex interplay of various risk factors. The results contribute valuable insights to the existing literature, guiding public health strategies and informing clinical interventions to mitigate the impact of congenital anomalies.

## 2. Objectives

The objectives of this study were to find socio-demographic profiles of newborns with congenital anomalies in Saudi Arabia, to determine the prevalence of newborns with congenital anomalies in Saudi Arabia, and to study the newborns with congenital anomalies and associated risk factors in Saudi Arabia.

## 3. Methodology

**Study Design and Settings:** This study adopts a descriptive cross-sectional record-based study design to investigate CA prevalence and associated factors among newborns delivered in a tertiary care maternity hospital—the Abha Maternity and Children Hospital (MCH), Abha, Kingdom of Saudi Arabia. This study encompassed newborns born between 2018 and 2022 at the Abha Maternity and Children Hospital. This study spanned from September 2023 to December 2023, providing a four-month window for data collection and analysis.

**Sampling Method:** Purposive sampling was employed, explicitly targeting newborns with congenital anomalies born between 2018 and 2022. Inclusion criteria involve children born with congenital anomalies in the study reference period, limited to those born at the Abha Maternity and Children Hospital. Exclusion criteria encompass children born without congenital anomalies, those whose mothers left the hospital against medical advice, and those referred to other medical centers after birth for various reasons. The entire population of children born with congenital anomalies and registered in the MCH Hospital over the past five years was included in this study.

**Method:** The data were collected by the researcher, who reviewed the hospital’s electronic medical records and used filters to filter the congenital anomalies cases delivered in this tertiary care maternity and children hospital; the researcher reviewed the selected medical record and electronic Google Forms used to enter the data, and this helped to efficiently collect and organize data by the researchers. **Data Collection Tool:** A self-designed and validated study tool was employed. The data collection tool comprises four major sections: socio-demographic details, family history, associated risk factors, and types of congenital anomalies.

**Statistical Analysis:** Collected data from Google Forms were downloaded into MS Office 2019 Excel spreadsheets and exported to IBM SPSS (Statistical Product and Service Solutions) version 21 for Windows employed for data analysis. Qualitative variables were expressed in proportions, and the chi-square test was applied to test hypotheses. The data were analyzed at a 95% confidence interval with 80% precision, and the significance level was set at *p* < 0.05.

**Ethics and Human Subjects Issues:** This study adhered to ethical standards, ensuring the confidentiality and privacy of the individuals’ data. The authors assured the hospital administration about data storage and security. The Research Ethics Committee (HAPO-06-B-001) at King Khalid University, Abha, Kingdom of Saudi Arabia, verified the study’s validated and ethical integrity and, after a careful review of the proposal, provided Institutional Ethical approval (ECM#2023-2509), dated 28 August 2023.

## 4. Results

Over the last five years (2018–2022) at the Abha Maternity and Children Hospital, there were a total of 14,664 births, with 14,647 (99.88%) being live births and 17 (0.11%) classified as stillbirths. Among the 14,664 deliveries, 472 (3.21%) reported congenital anomalies. In 2022 alone, out of 3158 births, 127 (4.02%) had congenital anomalies, contributing to an overall five-year prevalence of 3.21%.

Among the 472 neonates with congenital anomalies, 460 (97.5%) were live births, and 12 (1.5%) were stillbirths. Within the live birth group, 6 (1.3%) babies succumbed to severe congenital anomaly problems within their first month. Most malformations occurred in female babies (59.3%), while male babies accounted for 40.6%. Nearly 39.62% of babies were delivered preterm, and 60.4% were full-term, showing a significant *p*-value of 0.03. Half of the neonates with anomalies had normal birth weight (52.98%), and a considerable proportion (61.4%) of CA newborns had a history of parental consanguinity. However, this difference was not statistically significant (*p* > 0.44).

Regarding birth order, second-, third-, and fourth-order babies (28.2%, 24.6%, and 23.1%, respectively) presented with congenital anomalies, with no significant difference (*p* > 0.14). The occurrence of congenital anomalies in preterm neonates, at 39.6%, underscores the vulnerability of this population (Table 1).

Out of the 472 babies with anomalies, 428 (90.7%) had mothers aged below 40, and the majority (60.4%) of congenital anomaly newborns completed a full 9-month gestation period.

Congenital anomalies occur more frequently in babies born to fathers aged 40 and above (68%) compared to those with younger fathers. Still, the statistical difference between the two age groups is not significant (*p* > 0.08). Anomalies exhibit a positive association with maternal education, ranging from 3.7% for illiterate mothers to 30.1% for university graduates, but the difference is not statistically significant (*p* > 0.05). The prevalence of CA is higher among unemployed mothers (62.9%), those with antenatal medical problems (57.2%), and those exposed to passive smoking (55.7%). Only 66.7% of pregnant women attended ANC clinics; almost 99.8% of newborn anomalies were detected during antenatal screening. (Table 2).

Out of 472 congenital anomalies, 63.3% were linked to consanguineous marriages ranging from first-degree to third-degree relationships among parents. The most prevalent anomaly, accounting for nearly one-quarter of cases, was neural system defects (26.4%), primarily comprising neural tube defects (94.4%). Congenital heart diseases followed, representing one-fifth of anomalies (20.7%), with 33.3% and 30.0% associated with second-degree and third-degree consanguineous relations, respectively. Notably, over half of the cases of limb anomalies and transposition of the great vessels occurred in non-consanguineous marriages (Table 3).

Among 472 CA births, over three-fourths were linked to a family history of congenital anomalies; the difference is statistically significant (*p* < 0.001). Only a small number tested positive for COVID-19 during pregnancy, and calcium supplementation during antenatal care showed positive associations with cardiovascular system issues (79%) and neural tube defects (87.3%). No significant link was found between congenital anomalies, calcium supplementation, and COVID-19. Oro-facial clefts (60.0%) are commonly presented in newborns with passive smoking mothers. The difference is statistically significant (*p* < 0.009). Only 11 (2.3%) CA babies were linked to maternal active smoking. Advanced maternal age was linked to neural system defects, in particular neural tube defects (NTDs), while paternal age over 40 contributed most commonly to cardiovascular issues and NTDs. (Table 4).

Maternal medical issues during pregnancy exacerbated congenital anomalies such as NSDs (64%), CHDs (58%), renal anomalies (61%), limb issues (67%), and respiratory problems (68%) with statistically significant differences (*p* < 0.001). Medication use in pregnancy is often associated with specific congenital anomaly conditions, contributing to hip problems in 67% and renal agenesis in 64%. Contraceptive use is linked to hip problems in 37% and respiratory anomalies in 26%, showing statistical significance (*p* < 0.001). Notably, around 10% of individuals taking IFA supplementation experience anomalies, prompting further evaluation to determine whether this is coincidental or linked to early antenatal initiation (Table 5).

## 5. Discussion

The population in Saudi Arabia bears a considerable burden of congenital anomalies (CA), exhibiting a high rate of 32.1 per 1000 births or 1 in 32 births. In 2014, the WHO (World Health Organization) estimated that congenital malformation affects 1 in every 33 infants globally [11]. Over the last five years (2018–2022), the Abha Maternity and Children Hospital witnessed 3.21% of reported congenital anomalies. Notably, in 2022 alone, 4.02% exhibited congenital anomalies, reflecting a slight increase in prevalence compared to the five years prior. This temporal variation prompts further exploration into potential factors influencing congenital anomalies.

Similar comparable studies by Sallout B.I. et al. [1] and Ahmed M. Kurdi et al. [6] in Saudi Arabia also observed that the birth prevalence of congenital malformations was 34.57 per 1000 births and 41.2/1000 births, respectively. Another study by Sallout B. et al. [12] found a slightly higher percentage of 5.21% antenatal and 4.64% postnatal prevalence. This surpasses the rates observed in high-income countries by studies like EUROCAT (239/10,000 births) [13], BINOCAR (47.9/1000 births) [14], and the UK-based Bradford study (30.5/1000 births) [15]. Another study in the USA conducted by Parker S.E. et al. noted a 3% prevalence of CA [16]. Notably, this study’s prevalence rate is even higher than earlier reports in Saudi Arabia, which ranged from 170 to 465 per 10,000 live births [6,17,18]. However, caution is advised regarding some studies indicating higher rates, as they may overestimate the true prevalence by incorporating referrals from other institutions. The utilization of advanced ultrasound technology and the expertise of skilled practitioners contribute to the improved diagnosis of congenital anomalies. Heightened detection rates are attributed to a higher prevalence of these anomalies and advancements in diagnostic techniques.

The gender distribution of congenital anomalies among neonates revealed a higher incidence among female babies, constituting 59.3%, while male babies accounted for 40.6%. A similar previous study employed in Iran by Abdi-Rad I. et al. also noted that CA was predominant in female babies compared to male babies, and the findings were consistent with this study’s findings [19]. Another study by Madi S.A. et al., conducted in Kuwait, contradicted this study’s findings, indicating that congenital anomalies were predominant in male babies [20]. This gender discrepancy prompts a deeper investigation into the biological and environmental factors that might contribute to this observed pattern.

There is an intriguing association between gestational age and the occurrence of inborn anomalies among newborns; surprisingly, this study observed that most congenital malformations were associated with full-term babies and 39.6% of preterm births with congenital anomalies. Former parallel studies conducted in different parts of the globe, one in Morocco by Elghanmi et al., are consistent with this study’s findings [21]. Another one in India by Sachdeva S. et al. contradicts this study’s findings [22]. The differences in the findings may be due to the geographical and study settings. This finding underscores the vulnerability of preterm neonates to congenital anomalies, emphasizing the need for specialized care and attention to this population.

Birth weight also emerged as a risk factor for CA, with 53% of neonates with congenital anomalies having a normal birth weight. A study conducted in Morocco by Elghanmi et al. is inconsistent with this study [21]. El Koumi M.A. et al.’s study observed contrasting findings with this study’s findings [23]. These challenges conventional assumptions linking low birth weight to an increased risk of CA, prompting a reassessment of the factors contributing to these anomalies.

Exploring familial factors, this study delved into the family history of congenital anomalies and parental consanguinity, revealing that 63.4% of newborns with CA had consanguineous parents. The Sallout B.I. et al. [1] study observed a lesser percentage of consanguineous marriages among the parents of CA newborns. Consanguineous relationships account for 37.9% of the Saudi population. Another study by Taksande et al. in central India was consistent with this study’s findings that consanguinity nearly doubled the risk for congenital anomalies [24].

Examining the role of birth order, the study identified a pattern where second-order babies exhibited anomalies more frequently (28.2%), followed by third-order (24.6%) and fourth- (and above) order births (23.1%). A study by Hay S. et al. also determined that CA is directly proportional to birth order [25]. This trend prompts exploration into potential familial or genetic factors influencing congenital anomalies based on birth order.

Specifically, the reported incidence of neural tube defects (NTDs) in Saudi Arabia stands at 25% of all congenital anomalies. A study by Sachdeva S. et al. conducted in India [22] also aligns with this study’s findings, indicating that the most common (44.68%) CA was neural tube defects (anencephaly). Another survey by Abdi-Rad I. et al. in Iran noted that the most common CA was nervous system defects (52.65%) [19]. The incidence of NTDs is comparatively higher than in other studies in Kuwait (1.3/1000) [6] and Shiraz, Iran (1.6/1000) [19]. Folic acid deficiency is strongly associated with NTDs, and the supplementation of folic acid has led to a noteworthy reduction in incidence in Western countries.

Neonates born to mothers aged 20–30 and fathers over 40 exhibited higher rates of anomalies (54.2% and 68%, respectively). Many other similar studies are consistent with this study’s findings, such as the study by Sarkar S. et al. [26], whereas other similar studies, such as those by Dutta V. et al. and Suguna Bai N.S. et al., contradicted these findings [27].

The correlation between risk factors and congenital anomalies resonates with a family history of anomalies, with calcium intake during pregnancy resulting in cardiovascular system problems (77%), neural system defects (69.5%), and esophageal anomalies (86%). A study by Rayannavar et al. [28] revealed that 40% of congenital heart diseases are linked to hypocalcemia during pregnancy [28]. Passive smoking is more associated with oro-facial clefts (60%), emphasizing similarities to the research of Lie R.T. et al. conducted in Norway [29]. Medication usage during pregnancy contributes to specific congenital problems and also mirrors these findings. These parallels with the established literature validate the significance of the identified risk factors and their associations with specific congenital outcomes [30].

This study offers valuable perceptions, such as demographic distributions and associated risk factors for CA in neonates. Gender distribution, association with preterm births, birth weight considerations, and familial factors and birth order were considered when evaluating the contributions to congenital anomalies within this specific population. Genetic and environmental factors are essential in specific anomalies like neural tube defects and congenital heart diseases. Consanguineous marriages have a significant impact on particular anomalies. This finding emphasizes the need for genetic counselling and awareness programs for couples planning consanguineous marriages. The increased risks associated with family history, calcium intake, and pregnancy-induced hypertension reiterate the multifactorial nature of these anomalies. Medical issues during pregnancy, medication usage, contraceptive use, and iron and folic acid supplementation are potential contributors to specific congenital anomalies. These insights are crucial for healthcare practitioners in managing pregnancies with these risk factors.

## 6. Conclusions

This current research emphasizes significant connections between demographic factors and congenital anomalies in neonates. Noteworthy risk factors include preterm births, consanguineous marriages, mothers’ education and occupations, family history, maternal health issues, medication, and birth weight. These findings could inform MCH healthcare planning, facilitating targeted interventions and promoting 100% comprehensive antenatal screening. Proactive measures, such as prophylactic medication for high-risk individuals, are suggested to prevent malformations. Additionally, planning preventive strategies like pre-conceptional and premarital screening, counselling, and awareness programs are crucial to mitigate risks linked to preterm births as a result of consanguineous marriages. These insights enhance understanding and pave the way for prospective research with a substantial sample, aiding public health initiatives in minimizing congenital anomalies, reducing neonatal and infant mortality, and improving pregnancy outcomes.

## 7. Recommendations

Preconception counselling and premarital testing, especially for consanguineous married couples, can provide valuable information about potential risks and guide them to make informed family planning decisions. Strengthening antenatal education programs can raise awareness among expectant mothers about the impact of factors such as age, education, and employment status on neonatal health. Implementing community-based awareness programs can help to educate the population via measures discouraging consanguineous marriages, promoting the importance of a healthy lifestyle, promoting proper nutrition, and encouraging individuals to avoid known risk factors during pregnancy.

## 8. Limitations

The limited data on congenital newborns who were transferred out and those who lacked follow-up studies, coupled with this study’s sole reliance on one institution, may not accurately reflect the entire nation. Additionally, this study’s use of purposive sampling and a descriptive cross-sectional approach underscores the need for a large-scale prospective research effort to identify associated risk factors comprehensively. Such research can inform public health initiatives to reduce congenital-related neonatal mortality and enhance overall pregnancy outcomes.

## Figures and Tables

**Table 1:** The demographic profile of newborns with congenital anomalies.

		Gender of the Baby						
		Female		Male		Total		*p*-Value
		No	%	No	%	No	%	
Gestational age	Preterm	100	35.7%	87	45.3%	187	39.6%	0.03 ^$^
	Full-term	180	64.3%	105	54.7%	285	60.4%	
Newborn’s Birth Weight	<2.5 kg	110	39.3%	76	39.6%	186	39.4%	0.87 ^
	2.5 kg–3.5 kg	150	53.6%	100	52.1%	250	53.0%	
	>3.5 kg	20	7.1%	16	8.3%	36	7.6%	
Parents consanguineous	No	104	37.1%	78	40.6%	182	38.6%	0.44 ^
	Yes	176	62.9%	114	59.4%	290	61.4%	
Birth order	1st baby	68	24.3%	46	24.0%	114	24.2%	0.14 ^
	2nd baby	85	30.4%	48	25.0%	133	28.2%	
	3rd baby	65	23.2%	51	26.6%	116	24.6%	
	≥4	62	22.1%	47	24.5%	109	23.1%	
	Total	280	100.0%	192	100.0%	472	100.0%	

^$^—Significant; ^ = not significant.

**Table 2:** Demographic information and antenatal condition of parents and the gender of the baby.

		Gender of the Newborn						*p*-Value
		Female		Male		Total		
		No	%	No	%	No	%	
Age of the mother	<20 yrs.	52	18.6%	25	13.0%	77	16.3%	0.36 ^
	20–30 yrs.	149	53.2%	107	55.7%	256	54.2%	
	30–40 yrs.	52	18.6%	43	22.4%	95	20.1%	
	>40	27	9.6%	17	8.9%	44	9.3%	
Mother’s education	Illiterate	12	4.3%	6	3.1%	18	3.8%	0.5 ^
	Primary	27	9.6%	24	12.5%	51	10.8%	
	High School	98	35.0%	66	34.4%	164	34.7%	
	Intermediate	63	22.5%	34	17.7%	97	20.6%	
	University	80	28.6%	62	32.3%	142	30.1%	
Mother’s occupation	Business	51	18.2%	31	16.1%	82	17.4%	0.08 ^
	Government Employee	27	9.6%	33	17.2%	60	12.7%	
	Student	18	6.4%	15	7.8%	33	7.0%	
	Unemployed	184	65.7%	113	58.9%	297	62.9%	
Age of the father	<40	81	28.9%	70	36.5%	151	32.0%	0.08 ^
	>40	199	71.1%	122	63.5%	321	68.0%	
Antenatal medical problems for the mother	No	119	42.5%	83	43.2%	202	42.8%	0.8 ^
	Yes	161	57.5%	109	56.8%	270	57.2%	
Treatment for infertility	No	275	98.2%	185	96.4%	460	97.5%	0.2 ^
	Yes	5	1.8%	7	3.6%	12	2.5%	
History of smoking during pregnancy	Non-Smoker	119	42.5%	79	41.1%	198	41.9%	0.9 ^
	Current Smoker	6	2.1%	5	2.6%	11	2.3%	
	Passive	155	55.4%	108	56.3%	263	55.7%	
History of trauma during pregnancy	No	267	95.4%	174	90.6%	441	93.4%	0.04 ^$^
	Yes	13	4.6%	18	9.4%	31	6.6%	
Prenatal determination of congenital anomalies	No	88	31.4%	72	37.5%	160	33.9%	0.001 ^$^
	Yes	192	68.6%	120	62.5%	312	66.1%	
History of antenatal checkups	No	87	31.1%	70	36.5%	157	33.3%	0.2 ^
	Yes	193	68.9%	122	63.5%	315	66.7%	
	Total	280	100.0%	192	100.0%	472	100.0%	

^$^—Significant; ^ = not significant.

**Table 3:** Type of congenital anomalies associated with consanguineous marriages of parents.

Type of Congenital Anomaly	Degree of Parents Consanguineous									
	1st Degree		2nd Degree		3rd Degree		NA		Total	
	No	%	No	%	No	%	No	%	No	%
Abdominal wall defects	2	3.5%	13	22.4%	21	36.2%	22	37.9%	58	100%
Urinary system	6	21.4%	10	35.7%	7	25.0%	5	17.9%	28	100%
CHDs *	2	2.00%	33	33.00%	30	30.00%	35	35.00%	100	100%
Oro-facial clefts	3	7.5%	13	32.5%	13	32.5%	11	27.5%	40	100%
Respiratory system anomalies	1	1.8%	16	28.1%	23	40.4%	17	29.8%	57	100%
Limb defects	1	2.3%	4	9.3%	13	30.2%	25	58.1%	43	100%
NSDs #	5	4.0%	42	33.6%	39	31.2%	39	31.2%	125	100%
Ear, eye, face, and neck (minor)	11	24%	9	20%	14	30%	12	26%	46	100%
Multisystem anomalies	4	18%	7	32%	7	32%	4	18%	22	100%
Other (genital and facial)	1	3.6%	3	10.7%	1	3.6%	23	82.1%	28	100%

X^2^ = 71.1; df = 18, *p* < 0.001 Significant (yates’ correction) * Congenital heart defects; # Neural system defects.

**Table 4:** Distribution of type of congenital anomalies associated with risk factors.

		Abdominal Wall Defects		Urinary System		CHDs *		Oro-Facial Clefts		Respiratory Anomaly		Limb Defects		NSDs #		Other (Genital and Facial)		*p* Value
		No	%	No	%	No	%	No	%	No	%	No	%	No	%	No	%	
Gender of baby		Female	35	13%	16	6%	58	21%	25	9%	36	13%	26	9%	69	25%	15	0.99 ^
		Male	23	12%	12	6%	42	22%	15	8%	21	11%	17	9%	49	26%	13	
F/H of congenital anomalies	No	15	25.9%	5	17.9%	23	23%	7	17.5%	11	19.3%	18	41.9%	36	30.5%	24	85.7%	0.001 ^$^
	Yes	43	74.1%	23	82.1%	77	77%	33	82.5%	46	80.7%	25	58.1%	82	69.5%	4	14.3%	
COVID-19 during pregnancy	No	55	94.8%	26	92.9%	97	97%	37	92.5%	54	94.7%	42	97.7%	117	99.2%	18	64.3%	-
	Yes	3	5.2%	2	7.1%	3	3%	3	7.5%	3	5.3%	1	2.3%	1	0.8%	10	35.7%	
Received calcium during pregnancy	No	19	32.8%	0	0.0%	11	11%	12	30.0%	8	14.0%	4	9.3%	15	12.7%	18	64.3%	-
	Yes	39	67.2%	28	100.0%	89	89%	28	70.0%	49	86.0%	39	90.7%	103	87.3%	10	35.7%	
PIH ^##^	No	44	75.9%	22	78.6%	66	66%	25	62.5%	36	63.2%	36	83.7%	86	72.9%	12	42.9%	0.009 ^$^
	Yes	14	24.1%	6	21.4%	34	34%	15	37.5%	21	36.8%	7	16.3%	32	27.1%	16	57.1%	
Gestational diabetes	No	44	15%	19	7%	59	20%	27	9%	35	12%	19	7%	74	26%	11	4%	0.14 ^$^
	Yes	14	8%	9	5%	41	22%	13	7%	22	12%	24	13%	44	24%	17	9%	
Anomalies in mothers	No	52	89.7%	24	85.7%	70	70%	34	85.0%	50	87.7%	40	93.0%	107	90.7%	28	100.0%	0.001 ^$^
	Yes	6	10.3%	4	14.3%	30	30%	6	15.0%	7	12.3%	3	7.0%	11	9.3%	0	0.0%	
Smoking during pregnancy	No	25	43.1%	10	35.7%	35	35%	16	40.0%	18	31.6%	14	32.6%	68	57.6%	12	42.9%	-
	yes	2	3.4%	2	7.1%	1	1%	0	0.0%	0	0.0%	0	0.0%	6	5.1%	0	0.0%	
	Passive	31	53.4%	16	57.1%	64	64%	24	60.0%	39	68.4%	29	67.4%	44	37.3%	16	57.1%	
Mother’s age (yrs.)	<20	15	25.9%	6	21.4%	12	12%	6	15.0%	8	14.0%	5	11.6%	20	16.9%	5	17.9%	0.27 ^$^
	20–30	23	39.7%	15	53.6%	64	64%	23	57.5%	33	57.9%	23	53.5%	65	55.1%	10	35.7%	
	30–40	16	27.6%	5	17.9%	15	15%	8	20.0%	11	19.3%	13	30.2%	20	16.9%	7	25.0%	
	>40	4	6.9%	2	7.1%	9	9%	3	7.5%	5	8.8%	2	4.7%	13	11.0%	6	21.4%	
Father’s age (yrs.)	<40	22	37.9%	8	28.6%	22	22%	18	45.0%	20	35.1%	17	39.5%	35	29.7%	9	32.1%	0.16 ^$^
	>40	36	62.1%	20	71.4%	78	78%	22	55.0%	37	64.9%	26	60.5%	83	70.3%	19	67.9%	
History of antenatal checkups	No	15	10%	9	6%	36	23%	13	8%	20	13%	13	8%	32	20%	19	12%	0.008 ^$^
	Yes	43	14%	19	6%	64	20%	27	9%	37	12%	30	10%	86	27%	9	3%	
	Total	58	12%	28	6%	100	21%	40	8%	57	12%	43	9%	118	25%	28	6%	

* Congenital heart defects; # neural system defects; ^##^ PIH—pregnancy-induced hypertension. ^$^—Significant; ^ = not significant.

**Table 5:** Distribution of congenital anomalies related to issues during pregnancy.

During Pregnancy		Abdominal Wall Defects		Renal System		CHDs *		Oro-Facial Clefts		Respiratory Anomaly		Limb Defects		NSDs #		Other (Genital and Facial)		*p* Value
		No	%	No	%	No	%	No	%	No	%	No	%	No	%	No	%	
Medical issues	No	31	53%	11	39%	17	43%	45	45%	18	32%	14	33%	43	36%	23	82%	0.001 ^$^
	Yes	27	47%	17	61%	23	58%	55	55%	39	68%	29	67%	75	64%	5	18%	
Medication	No	28	48%	10	36%	16	40%	50	50%	23	40%	15	35%	49	42%	24	86%	0.001 ^$^
	Yes	30	52%	18	64%	24	60%	50	50%	34	60%	28	65%	69	59%	4	14%	
Contraceptive usage	No	47	81%	26	93%	28	70%	79	79%	42	74%	27	63%	94	80%	28	100%	0.005 ^$^
	Yes	11	19%	2	7%	12	30%	21	21%	15	26%	16	37%	24	20%	0	0%	
X-ray/CT exposure	No	50	86%	24	86%	30	75%	96	96%	49	86%	36	84%	110	93%	18	64%	0.001 ^$^
	Yes	8	14%	4	14%	10	25%	4	4%	8	14%	7	16%	8	7%	10	36%	
IFA ^##^	No	49	85%	27	97%	29	73%	95	95%	51	90%	41	95%	106	90%	13	46%	-
	Yes	9	16%	1	4%	11	28%	5	5%	6	11%	2	5%	12	10%	15	54%	
	Total	58	100%	28	100%	40	100%	100	100%	57	100%	43	100%	118	100%	28	100%	

* Congenital heart defects; # Neural system defects; ^##^ Iron and folic acid; ^$^—Significant.

## Data Availability

The datasets used and analyzed during this current study are available from the corresponding author upon reasonable request and will be provided after masking the identity of the individuals. Data available on request due to restrictions eg privacy and ethical.
